# Effect of Nedocromil Sodium on Polymorphonuclear Leukocyte Plasma Membrane

**DOI:** 10.1155/S0962935194000700

**Published:** 1994

**Authors:** A. Kantar, N. Oggiano, P. L. Giorgi, G. V. Coppa, R. Gabbianelli, S. Bruni, F. M. Cutrona, R. Fiorini

**Affiliations:** 1 Department of Paediatrics University of Ancona Via Corridoni II Ancona I-60123 Italy; 2 Department of Biochemistry University of Ancona Via Corridoni II Ancona I-60123 Italy

## Abstract

The effect of nedocromil sodium on the plasma membrane fluidity of polymorphonuclear leukocytes (PMNs) was investigated by measuring steady-state fluorescence anisotropy of 1-[4-trimethylammonium-phenyl]-6-phenyl- 1,3,5-hexatriene (TMA-DPH) incorporated in the membrane. Our results show that nedocromil sodium 300 μM significantly decreased membrane fluidity of PMNs. The decrease in membrane fluidity of PMNs induced by fMLP was abolished in the presence of nedocromil sodium. These data suggest that nedocromil sodium interferes with the plasma membranes of PMNs and modulates their activities.


Mediators of Inflammation 3, S21–S24 (1994)

MOIA3

### Effect of nedocromil sodium on polymorphonuclear leukocyte plasma membrane

A. Kantar,[1,CA] N. Oggiano,[1] P. L. Giorgi,[1] G. V. Coppa,[1] R. Gabbianelli,[1] S. Bruni,[1] F. M. Cutrona[1] and R. Fiorini[2]

Departments of [1]Paediatrics and [2]Biochemistry, University of Ancona, Via Corridoni II, I-60123 Ancona, Italy

[CA] Corresponding Author

**The effect of nedocromil sodium on the plasma membrane fluidity of polymorphonuclear leukocytes (PMNs) was investigated by measuring steady-state fluorescence anisotropy of 1-[4-trimethylammonium-phenyl]-6-phenyl-1,3,5-hexatriene (TMA-DPH) incorporated in the membrane. Our results show that nedocromil sodium 300 μM significantly decreased membrane fluidity of PMNs. The decrease in membrane fluidity of PMNs induced by fMLP was abolished in the presence of nedocromil sodium. These data suggest that nedocromil sodium interferes with the plasma membranes of PMNs and modulates their activities.**

**Key words**: Chemiluminescence, Fluorescence, Membrane, Nedocromil, Polymorphonuclear leukocyte

### Introduction

In response to inflammatory stimuli, a rapid and often massive influx of polymorphonuclear leukocytes (PMNs) can be driven to the inflamed tissues. This is particularly true in the lung, where PMNs traffic can be facilitated both by a rather thin barrier separating the alveolar space from the capillary lumen, and by the sequestration of PMNs in the pulmonary vasculature.[1] Although PMNs are probably not the dominant cells in asthma, they may contribute to the inflammatory process underlying the disease.[2] A series of membrane receptors have been identified on the surface of PMNs. These receptors constitute the link between PMNs and their environment, and modulate PMNs functions, including adherence, migration, degranulation, and respiratory burst.[1]

There is little argument that products of activated PMNs are tissue-damaging. In a variety of experimental models of inflammation, depletion of PMNs attenuates the intensity of tissue damage.[3] PMNs have tremendous potential to generate inflammation by releasing lysosomal enzymes, oxygen reactive species, histamine-releasing factor and inflammatory mediators.[4]

Airway inflammation has emerged as an important contributor to the mechanisms of asthma. Furthermore, the presence of airway inflammation is present even in the absence of severe symptoms. In asthmatics, nedocromil sodium has anti-inflammatory properties *in vitro*, as evidenced by inhibition of inflammatory cell activation and mediators release.[5] In this study, the effect of nedocromil sodium on membrane fluidity of PMNs was investigated using fluorescence technique.

### Materials and Methods

*Preparation of PMNs:* Blood samples were obtained from ten healthy donors and PMNs were isolated using a Mono-Poly Resolving Medium (ICN Biomedicals, Milan, Italy) as previously described.[6] Cells were resuspended in Krebs Ringer Phosphate solution (KRP), supplemented with 5 mM glucose.

*Chemiluminescence measurements:* Luminol-amplified chemiluminescence (CL) was measured in an AutoLumat LB 953 (Berthold Co., Wilbad, Germany) and PMNs in the presence or absence of nedocromil sodium 300 μM were activated by addition of *N*-formyl-methionyl-leucyl-phenylalanine (fMLP) (Sigma Chemical Co., St Louis, MO, USA) ($10^{-7}$ M) or phorbol myristate acetate (PMA) (Sigma Chemical Co.) $3 \times 10^{-4}$ M, as previously described.[7] Nedocromil sodium was kindly donated by Fisons plc, Pharmaceutical Division (Loughborough, Leics, UK).

*Fluorescence measurements:* Steady-state fluorescence anisotropy ($r_s$) measurements were performed at 37°C with a Perkin Elmer Spectrofluorimeter MPF-66, equipped with a Perkin Elmer 7300 Personal Computer for data acquisition and elaboration, using 1-[4-trimethylammoniumphenyl]-6-phenyl-1,3,5-hexatriene (TMA-DPH) (Molecular Probes, Inc., Eugene, OR, USA) as hydrophobic fluorescent probe at a final concentration of $10^{-6}$ M as previously described.[8] The computer program calculated $r_s$ by using the expression $(I_{\parallel} - I_{\perp} \times g)/(I_{\parallel} + 2I_{\perp} \times g)$ where $g$ is an instrument correction factor, $I_{\parallel}$ and $I_{\perp}$ are, respectively, the emission intensities with polarizers parallel and perpendicular to the direction of polarized light.[9] Labelling was carried out in the dark and

© 1994 Rapid Communications of Oxford Ltd

$r_s$ values were determined before and after addition of nedocromil sodium (300 μM).

The significance of values obtained was calculated using Student's *t*-test.

### Results

*Chemiluminescence studies:* When fMLP was added to PMNs, a bimodal response was obtained. The first peak appeared after approximately 1 min and the second peak occurred after 3–5 min. When PMNs were exposed to fMLP in the presence of nedocromil sodium (300 μM), a significant inhibition of both peaks was observed (Fig. 1). This inhibition was dose-dependent (data not shown). When PMNs were activated with PMA, a peak CL value was obtained 15–17 min after activation. No significant inhibition was observed in the presence of nedocromil 300 μM.

*Fluorescence study:* The background fluorescence of PMNs was checked before each measurement and was less than 0.1% of the fluorescence when TMA-DPH was added. In basal conditions, without stimulation, the $r_s$ of TMA-DPH incorporated in plasma membrane of PMNs was stable and did not show significant changes ($p > 0.5$) for 30 min after addition of TMA-DPH. In the absence of nedocromil, fMLP induced a significant and time-dependent increase in $r_s$ value (Fig. 2). Nedocromil sodium induced a significant ($p < 0.001$) and stable increase in the $r_s$ value (Fig. 3). The addition of fMLP to PMNs treated with nedocromil sodium abolished the effect of fMLP on PMNs plasma membrane (data not shown).

### Discussion

Nedocromil sodium has been shown to inhibit chemotaxis of human PMNs induced by zymosan activated serum, PAF, leukotriene B4 or fMLP.[10–12] Moreover, it inhibits the respiratory burst of PMNs stimulated with PAF or fMLP.[12,13]

The N-formyl peptide receptor of human PMNs is an integral membrane protein. Agonist binding to this chemoattractant receptor results in a variety of PMNs activities including chemotaxis, adhesion, superoxide production and secretion of hydrolytic enzymes.[14]

The signal transduction cascade initiated by occupancy of N-formyl peptide receptors is a complex spatial and temporal orchestration of biochemical events mediated by specific molecules. Occupancy of the chemoattractant receptor with ligand results in the formation of a ligand-receptor complex. This complex interacts with guanyl nucleotide binding protein (G), resulting in the formation of activated G protein, which then activates phospholipase C (PLC) in the plasma membrane. PLC activation results in phosphatidylinositol (PI) hydrolysis to generate 1,4-5-inositoltriphosphate (IP3) and diacylglycerol (DAG). IP3 induces release of $Ca^{2+}$ from intracellular stores, which, in concert with DAG, activate and cause the translocation of intracellular protein kinase C (PKC) to the plasma membrane. These events results in phosphorylation of membrane proteins.[14,15] The consequent biochemical events converge in the assembly and activation of the complex enzymatic system NADPH-oxidase.

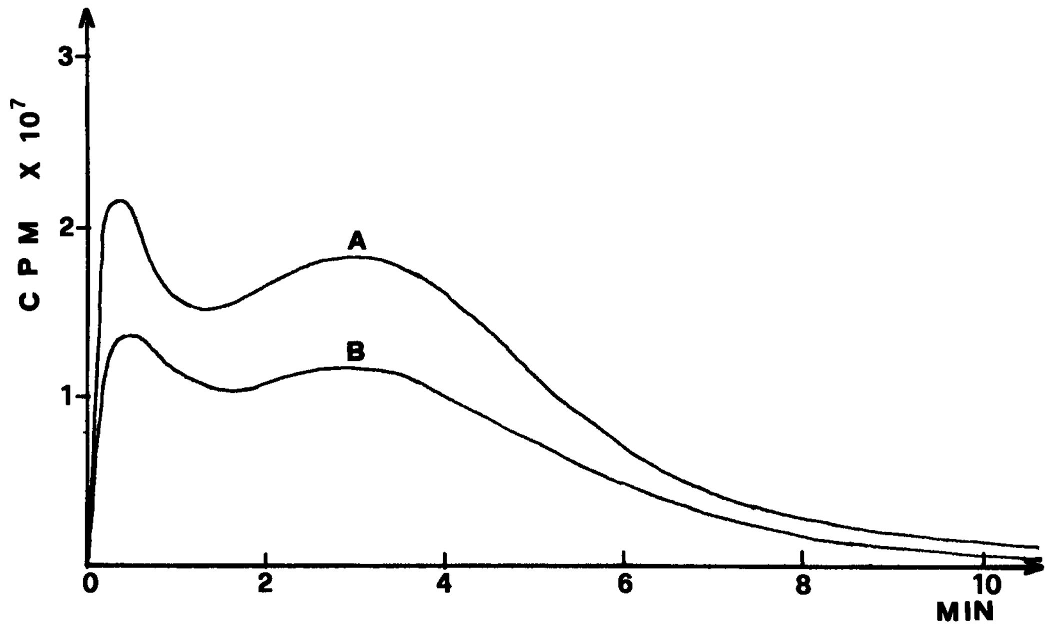

FIG. 1. Time-course of luminol-amplified CL of PMNs stimulated with fMLP in absence (A) or presence (B) of nedocromil sodium (300 μM). CL was measured as counts per minute (cpm). Data are the mean of ten samples.

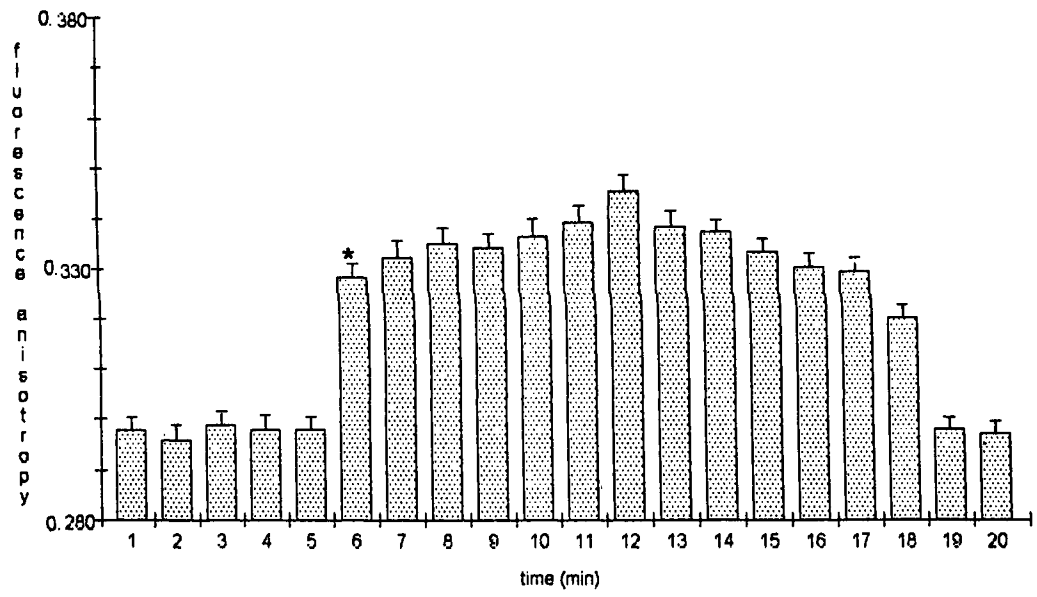

FIG. 2. Fluorescence anisotropy $r_s$ of TMA-DPH in PMNs plasma membrane before and after addition of fMLP ($10^{-7}$ M). fMLP induced a time-dependent and significant ($p < 0.001$) increase in $r_s$ value. Data are the mean (+2SD) of ten samples. (*) Addition of fMLP.

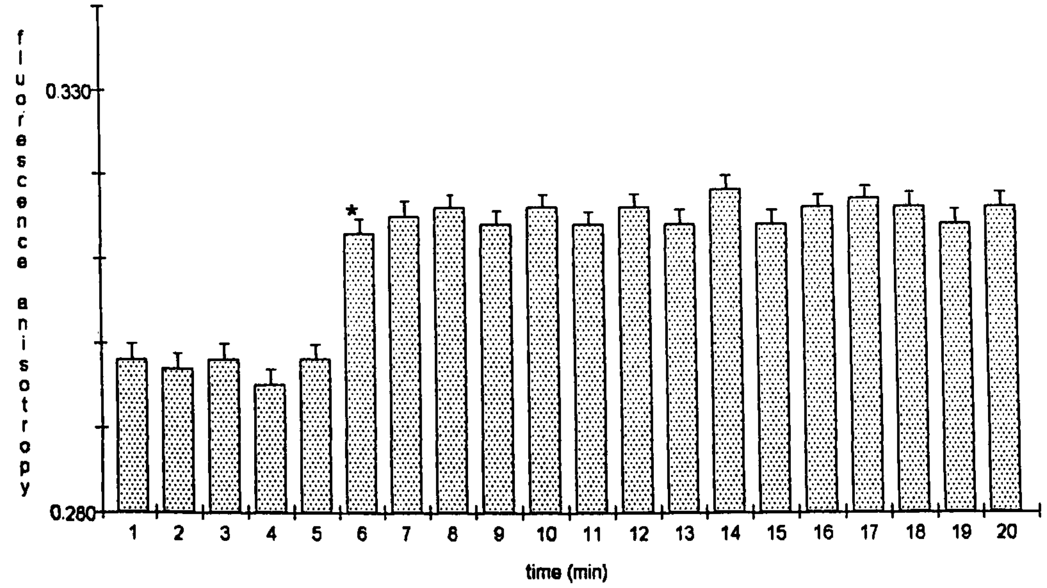

FIG. 3. Fluorescence anisotropy $r_s$ of TMA-DPH in PMNs plasma membrane before and after addition of nedocromil sodium (300 μM). Nedocromil induced a stable and significant ($p < 0.001$) increase in $r_s$ value. Data are the mean (+2SD) of ten samples. (*) Addition of nedocromil.

There is now evidence to indicate that each integral membrane protein interacts with its neighbouring 'boundary' lipids in a specific manner.[16] Because of the pronounced effect of different lipids on the operation of several membrane proteins and membrane processes, it is likely that changes in the microenvironment of membrane proteins, due to changes in the lateral organization of lipids, could be used for regulatory purposes.[17] Alterations in the composition of this lipid boundary may lead to changes in enzyme activity, as well as to changes in ligand specificity and affinity, as shown recently for the vitronectin receptor.[18]

Membrane fluidity is a complex physicochemical feature which depends upon mobility and on the order of membrane constituents.[19] TMA-DPH has been widely used for studying membrane structure and fluidity because of its advantageous structural and photophysical properties.[20] TMA-DPH incorporates into the membrane, but remains at the lipid–water interface region because of its cationic residue. TMA-DPH $r_s$ reflects the packing of membrane lipid fatty acid chains, and can be related to the order parameter S, if certain precautions are taken.[21] Lipid fluidity may be defined as the reciprocal of the lipid structural order parameter S,[21] and thus an increase of

TMA-DPH $r_s$ value corresponds to a decrease in membrane fluidity. In our study, it was observed that nedocromil sodium induced a significant increase in the $r_s$ value of TMA-DPH in PMNs plasma membrane. This indicates a decrease in membrane fluidity. To investigate whether these changes could influence formyl peptide receptors function, the TMA-DPH $r_s$ value was evaluated after the addition of fMLP to PMNs incubated with nedocromil sodium. Our results demonstrate that nedocromil abolished the previously reported decrease in membrane fluidity induced by fMLP.[9] This is in accordance with the recent data reported by Peroni *et al.* which demonstrated that nedocromil sodium decreases the binding of fMLP to PMNs.[22]

To obtain more evidence that nedocromil sodium (300 μM) can influence plasma membrane activity, the response of nedocromil-treated PMNs to fMLP and PMA was investigated. These activators of the respiratory burst employ different pathways during the stimulation of PMNs. PMA activates the PKC, directly bypassing all the previously mentioned biochemical pathways employed by fMLP.[23] Since nedocromil sodium did not induce changes in PMA stimulation, it is likely that the changes induced by nedocromil sodium occur prior to PKC activation. These results are in accordance with previous studies that reported no influence of nedocromil on PMNs activated with PMA, whereas a significant inhibition on PMNs stimulated with fMLP or PAF occurred.[12,13] Our data suggest that nedocromil sodium interferes with the plasma membranes of PMNs, and hence influences their functional activities.

### References

1. Sibille Y, Marchandise FX. Pulmonary immune cells in health and disease: polymorphonuclear neutrophils. *Eur Respir J* 1993; **6**: 1529–1543.
2. Shock A, Laurent GJ. Leukocytes and pulmonary disorders: mobilization, activation and role in pathology. *Mol Aspects Med* 1990; **11**: 425–526.
3. Ward PA, Mulligan MS. Leukocyte oxygen products and tissue damage. In: Jesaitis AJ, Dratz EA, eds. *Molecular Basis of Oxidative Damage by Leukocytes*. Boca Raton, FL: CRC Press, 1992; 139–147.
4. Henson PM, Johnston RB. Tissue injury in inflammation. Oxidants, proteinases and cationic proteins. *J Clin Invest* 1987; **79**: 669–674.
5. Brogden RN, Sorkin EM. Nedocromil sodium: an update review of its pharmacological properties and therapeutic efficacy in asthma. *Drugs* 1993; **45**: 693–715.
6. Kantar A, Wilkins G, Swoboda B, *et al.* Alterations of the respiratory burst of polymorphonuclear leukocytes from diabetic children. *Acta Paed Scand* 1990; **79**: 535–541.
7. Kantar A, Oggiano N, Gabbianelli R, Giorgi PL, Biraghi M. Effect of imidazole salicylate on the respiratory burst of polymorphonuclear leukocytes. *Curr Therapeut Res* 1993; **54**: 241–247.
8. Kantar A, Giorgi PL, Curatola G, Fiorini R. Alterations in membrane fluidity of diabetic polymorphonuclear leukocytes. *Biochem Med Metabol Biol* 1991; **46**: 422–426.
9. Fiorini R, Curatola G, Bertoli E, Giorgi PL, Kantar A. Changes of fluorescence anisotropy in plasma membrane of human polymorphonuclear leukocytes during the respiratory burst phenomenon. *FEBS Lett* 1990; **273**: 122–126.
10. Bruijnzeel PLB, Warringa RA, Kok PT. Inhibition of platelet-activating factor- and zymosan-activated serum-induced chemotaxis of human neutrophils by nedocromil sodium, BN 52021 and sodium cromoglycate. *Br J Pharmacol* 1989; **97**: 1251–1257.
11. Bruijnzeel PLB, Warringa RA, Kok PT, Hamelink ML, Kreukniet H, Koenderman L. Effects of nedocromil sodium on *in vitro* induced migration, activation and mediators release from human granulocytes. *J Allergy Clin Immunol* 1993; **92**: 159–164.
12. Rubin RP, Thompson RH, Naps MS. Differential inhibition by nedocromil sodium of superoxide generation elicited by platelet activating factor in human neutrophils. *Agents Actions* 1990; **31**: 237–242.
13. Peroni DG, Piacentini GL, Melotti P, Boner AL. Inhibition of nedocromil sodium superoxide production in human neutrophils. A preliminary communication. *Drug Invest* 1992; **4**: 386–390.
14. Omann GM, Allen RA, Bokoch GM, Painter RG, Traynor AE, Sklar LA. Signal transduction and cytoskeletal activation in the neutrophil. *Physiol Rev* 1987; **67**: 285–322.
15. Jeasitis AJ, Allen RA. Activation of the neutrophil respiratory burst by chemoattractants: regulation of the N-formyl peptide receptor in the plasma membrane. *J Bioenerg Biomemb* 1988; **20**: 679–707.
16. Watts A. Membrane structure and dynamics. *Curr Opin Cell Biol* 1989; **1**: 691–700.
17. Kinnunen PKJ. On the principles of functional ordering in biological membranes. *Chem Phys Lipids* 1991; **57**: 375–399.
18. Confronti G, Zanetti A, Pasquali-Ronchetti I, Quaglino D, Neyroz P, Dejana E. Modulation of vitronectin receptor by membrane lipid composition. *J Biol Chem* 1990; **265**: 4011–4019.
19. Shinitzky M. Membrane fluidity and cellular functions. In: Shinitzky M, ed. *Physiology of Membrane Fluidity*. Boca Raton, FL: CRC Press, 1984; 1–51.
20. Lentz BR. Membrane 'fluidity' as detected by diphenylhexatriene probes. *Chem Phys Lipids* 1989; **50**: 171–190.
21. Van Blitterswijk WJ, Van Hoeven RP, Van Der Meer BW. Lipid structural order parameters (reciprocal of fluidity) in biomembranes derived from steady-state fluorescence polarization measurements. *Biochim Biophys Acta* 1981; **644**: 323–332.
22. Peroni DG, Melotti P, Piacentini GL, Bonizzato C, Boner AL. Effects of nedocromil sodium on the binding of *N*-formyl-methionyl-leucyl-phenylalanine in human neutrophils. *Agents Actions* 1992; **36**: 212–214.
23. Lambeth JD. Activation of the respiratory burst oxidase in neutrophils: on the role of membrane-derived second messengers, $Ca^{++}$, and protein kinase C. *J Bioenerg Biomemb* 1988; **20**: 709–733.
